# ASO Author Reflections: Tailored Robotic Transmesocolic Approach for Left Retroperitoneal Lesions

**DOI:** 10.1245/s10434-026-20106-4

**Published:** 2026-06-27

**Authors:** Koichi Tomita, Naruhiko Ikoma

**Affiliations:** Department of Surgical Oncology, The University of Texas MD Anderson Cancer Center, Houston, TX USA

Resection of small retroperitoneal lesions, including nodal metastases, is well suited to minimally invasive surgery. Although robotic surgery provides stable three-dimensional vision and wristed instrumentation, it can be less efficient than laparoscopy for wide, dynamic mobilization of organs, such as the colon and small intestine, because standard solo robotic surgery provides fewer instruments for simultaneous traction and countertraction and a more restricted working field than laparoscopy. In patients with bowel distension or obesity, robotic mobilization of the colon and small intestine may therefore increase operative time and add complexity.

To address this limitation, we present a robotic transmesocolic approach that allows direct access to the left retroperitoneum without mobilizing the colon.^1^ Because retroperitoneal anatomy differs between the right and left sides, this article complements our previous description of the right-sided approach by demonstrating the left-sided technique.^2^ The key technical step is to incise the peritoneum at the ligament of Treitz and extend the dissection leftward along the inferior border of the pancreas. This creates a short, direct route through the mesentery to the region around the left renal vein and renal artery. Recognition of key anatomic landmarks, including the ligament of Treitz, pancreas, inferior mesenteric vein, left renal vein, and left renal artery, provides reliable orientation and enables precise and safe access to the left retroperitoneum, even in obese patients with thick mesenteric or retroperitoneal fat.

For retroperitoneal lesions located more caudally than the left renal vessels, a conventional approach involving left colon mobilization may remain useful, and the optimal approach can be selected according to the location of the target lesion and intra-abdominal conditions in each patient. We are accumulating additional cases using this approach, which may broaden the role of tailored robotic transmesocolic access for selected retroperitoneal lesions.
